# Influenza-Related Communication and Community Mitigation Strategies: Results From the 2015 Pandemic Influenza Readiness Assessment

**DOI:** 10.1177/1524839919826582

**Published:** 2019-02-17

**Authors:** Rupesh I. Naik, Sara J. Vagi, Amra Uzicanin, Stephanie A. Dopson

**Affiliations:** 1 Centers for Disease Control and Prevention, Atlanta, GA, USA; 2 Commissioned Corps of the United States Public Health Service, Atlanta, GA, USA

**Keywords:** community intervention, crisis and emergency risk communication for pandemic influenza, disaster and emergency preparedness, program planning and evaluation

## Abstract

Emergence of a novel infectious disease, such as pandemic influenza, is the one global crisis most likely to affect the greatest number of people worldwide. Because of the potentially severe and contagious nature of influenza, a rapid multifaceted pandemic response, which includes nonpharmaceutical interventions (NPIs) and effective strategies for communication with the public are essential for a timely response and mitigating the spread of disease. A web-based questionnaire was administered via email in July 2015 to 62 Public Health Emergency Preparedness (PHEP) directors across jurisdictions that receive funding through the Centers for Disease Control and Prevention PHEP cooperative agreement. This report focuses on two modules: Public Information and Communication and Community Mitigation. Consistent and targeted communication are critical for the acceptability and success of NPIs. All 62 jurisdictions have developed or are in the process of developing a communications plan. Community-level NPIs such as home isolation, school closures, and respiratory etiquette play a critical role in mitigating the spread of disease. Effective, ongoing communication with the public is essential to ensuring wide spread compliance of NPI’s, especially among non–English-speaking populations. Planning should also include reaching vulnerable populations and identifying the correct legal authorities for closing schools and canceling mass gatherings.

## Background

Emergence of a novel infectious disease, such as pandemic influenza, is the one global crisis most likely to affect the greatest number of people worldwide (Centers for Disease Control and Prevention [CDC], 2004). Because of the potentially severe and contagious nature of influenza, a rapid multifaceted pandemic response, which includes nonpharmaceutical interventions (NPIs) and effective strategies for communication with the public are essential for a timely response and mitigating the spread of disease (Association of State and Territorial Health Officials [ASTHO], 2008; CDC, 2018a, 2018b, 2018c, 2018d; Homeland Security Council, 2005; Germann, Kadau, Longini, & Macken, 2006; Taubenberger & Morens, 2006).

Community mitigation measures such as NPIs are measures that people and communities can take to slow the spread of infectious diseases. During an evolving pandemic, NPIs are the first line of defense before a vaccine is available (Qualls et al., 2017). While a number of NPIs are routinely endorsed (e.g., staying home when sick, hand hygiene), additional community-level NPIs aimed at reducing exposure may be recommended during pandemics (e.g., school closures, social distancing, and/or cancelling mass gatherings; Qualls et al., 2017). These community-level NPIs play a crucial role in slowing the spread of infection. Modeling and historical studies (Ferguson & Cummings, 2006; Markel, 2004, 2007) along with controlled studies (Aiello, 2010; Qualls et al., 2017) have suggested NPI’s are especially effective when implemented in conjunction with pandemic vaccination and other pharmaceutical interventions. However, the effectiveness of NPIs depends largely on appropriate and rapid dissemination of guidance and public compliance in an evolving pandemic; how messages are received, understood, and accepted by communities is key to successful implementation of NPIs. Therefore, prepandemic planning combined with instructions and information about NPIs are a critical component of public messaging before, during, and after an outbreak (Aiello, 2010; ASTHO, 2008; Qualls et al., 2017; U.S. Department of Health and Human Services [DHHS], 2005).

As described in the *Preparedness and Response Framework for Influenza Pandemics*, a pandemic has six intervals of activity: investigation, recognition, initiation, acceleration, deceleration, and preparation (Holloway, Rasmussen, Zaza, Cox, & Jernigan, 2014). The intervals are further stratified into eight domains, two of which are risk communication and community mitigation. These domains have specific roles during each interval of an influenza pandemic. For example, during the early part of the acceleration interval (consistently increasing rate of pandemic influenza cases), and based on pandemic severity, jurisdictions may consider whether enacting NPIs, such as school closures and workplace social distancing measures, may be appropriate for the response. In this article, we describe pandemic influenza preparedness at state, local, and territorial health departments. We also discuss evaluation of communication strategies and mitigation plans to trigger and implement NPIs by public health agencies and key audiences.

## Method

### Participants and Procedures

A web-based questionnaire was administered via email in July 2015 to 62 Public Health Emergency Preparedness (PHEP) directors across jurisdictions that receive funding through the CDC PHEP cooperative agreement. PHEP recipients include all public health departments in the 50 U.S. states, eight U.S. territories and freely associated states (Puerto Rico, U.S. Virgin Islands, American Samoa, Commonwealth of the Northern Mariana Islands, Guam, Republic of the Marshall Islands, Republic of Palau, and the Federated States of Micronesia), and four local jurisdictions (Chicago, IL; Los Angeles County, CA; New York, NY; and Washington, DC). A follow-up email was sent specifically to the identified experts requesting they complete the web-based questionnaire within 4 weeks; reminder emails were sent 7 and 14 days later to maximize response rates. Respondents for the Public Information and Communication module included eight PHEP directors, 32 Public Information Officers (PIOs), 14 Public Information Staff members, and eight “others” such as program managers and subject matter experts. The Community Mitigation module was answered by 17 PHEP directors, 27 epidemiologists, and 18 others such as program managers, nurses, and subject matter experts. Data were collected under OMB Approval Number 0920-0879. Descriptive analyses were conducted using SAS Version 9.3 (Cary, NC) and Microsoft Excel.

### Measures

The questionnaire addressed seven areas critical to pandemic planning, including *Epidemiology and Laboratory, Community Mitigation, Public Informa-tion and Communication, Medical Care and Counter-measures, Health Care Systems, Public Health Preparedness and Immunization Workforce*, and *Vaccination Planning*. Each module described a pandemic influenza scenario and assumptions that respondents were directed to consider when answering the questions.

This report focuses on two modules: *Public Information and Communication* and the *Community Mitigation*. The *Public Information and Communication* module assessed communication strategies between public health agencies and key audiences including the media, general public, and vulnerable populations. Vulnerable populations include those groups likely to be disproportionately affected during a pandemic such as economically disadvantaged persons, elderly, non-English speakers, and those with chronic health conditions. The module aimed to determine the extent to which public health agencies can effectively contact and influence diverse audiences with timely, accurate, and credible information about a pandemic influenza threat and promote protective actions. Question topics included communication planning, promotion of NPIs, vaccine clinics, and availability of antiviral medications, communications personnel, and communication channels. The *Community Mitigation* module assessed plans to recommend and implement NPIs during the earliest stages of an influenza pandemic. Specifically, triggers for implementing NPIs and plans for school and mass-gathering closures were addressed. Descriptive statistics for key questions from Community Mitigation and Public Information Communications modules were conducted. All percentages are based on a denominator of 62. Population and jurisdictional information were collected from the 2010 U.S. Census. Data were obtained from the World Health Organization’s Global Health Observatory Data Repository for territories and freely associated states when data were unavailable in the U.S. Census (U.S. Census Bureau, 2010). The total population covered by this study according to these sources is 313,023,593.

## Results

### Public Information and Communication

The response rate for both modules was 100%. Fifty-four (87%) jurisdictions had developed a communications plan for novel influenza outbreaks. The remaining jurisdictions, comprising 13% of the study population, reported that they were in the process of developing a plan. Ninety-seven percent of jurisdictions identified and trained key staff to serve as a spokesperson(s) during a novel influenza threat.

Reaching vulnerable populations is a specific focus of pandemic planning. Fifty-two (84%) of respondents have specific plans to reach vulnerable populations; the remaining jurisdictions representing 17% of the study population do not have plans for vulnerable populations. Table 1 highlights communication plan elements and reaching vulnerable population elements among jurisdictions with plans in place.

**Table 1:** Jurisdictions’ Reporting on Elements in Communication Plans and Reaching out to Vulnerable Populations (*N* = 62)

Element	% of Jurisdictions
Communication plan element	
Channels of communication	81
Goal and objectives	77
Strategies	77
Target audience	74
Tactics	71
Evaluation	39
Other	23
Vulnerable population element	
Securing translation services	79
Partnerships with agencies who serve vulnerable populations	77
Strategies for reaching vulnerable populations	71
Identification of non-English languages	69

All 62 jurisdictions established two-way channels for communicating with staff, partner organizations, the media, the general public, and other key audiences. Communication channels included conference call lines (97%), hotlines (84%), web conferencing capabilities (76%), and other types of two-way channels such as social media and email (42%); 84% had access to ≥3 of these communication channels. All but one jurisdiction developed a process for clearing the release of public information agreed upon by appropriate subject matter experts.

### Community Mitigation

All respondents incorporated a set of assumptions and triggers for implementing NPIs into pandemic influenza response plans. Thirty-two (52%) jurisdictions deemed themselves to have fully addressed assumptions and triggers in plans. These jurisdictions comprise 67% of the study population. Thirty (48%) jurisdictions have *partially* addressed assumptions and triggers, covering 33% of the study population. Respondents indicated that factors such as severity of illness (95%), transmissibility (94%), and populations most affected (90%) would be considered in choosing and triggering NPI implementation. Other important factors cited for introducing NPI interventions were CDC and subject matter expert recommendations, geographical spread of the disease, disease impact in relation to available mitigation resources, and vaccine availability.

During an influenza pandemic, CDC may recommend temporarily closing schools (child care facilities, K–12 schools, and colleges and universities) and canceling mass gatherings in affected jurisdictions (Qualls et al., 2017). As shown in Table 2, among the 62 jurisdictions surveyed, child care and K–12 facilities were more likely to implement recommendations for closure than colleges and universities, or mass gatherings. Additionally, child care and K–12 can close more quickly when necessary compared with the closure of colleges and the cancellation of mass gatherings.

**Table 2:** Child Care and K–12 Facilities Are More Likely to Implement Recommendations for Closure Due to Pandemic Influenza, United States,^a^ 2015 (*N* = 62)

Factors	Child Care Facilities, %	K–12, %	Colleges and Universities, %	Mass Gatherings, %
Likelihood of implementation of recommendations for closure				
Very likely/likely	82	85	66	52
Somewhat likely	10	8	19	32
Not at all likely	3	3	3	3
Unsure/do not know	5	3	10	13
Authorization needed to cancel schools or mass gatherings				
Do not need	24	23	23	21
Need and have	65	68	65	66
Need but DO NOT have	11	10	13	13
How long will it take to cancel schools or mass gatherings				
≤3 days	90	92	76	77
4-7 days	5	5	15	15
>1 week	3	2	3	6
Jurisdiction would not close schools	0	0	2	0

a United States refers to all public health departments in the 50 states; Puerto Rico; U.S. Virgin Islands; American Samoa; Commonwealth of the Northern Mariana Islands; Guam; Republic of the Marshall Islands; Republic of Palau; the Federated States of Micronesia, Chicago, IL; Los Angeles County, CA; New York, NY; and Washington, DC.

Most jurisdictions reported they have (or do not need) legal authority to close these institutions; however, nine jurisdictions (15%) do not have the authority necessary to temporarily close child care facilities, K–12 schools, colleges/universities, or cancel mass gatherings. Of these nine jurisdictions, seven are states, and two are territories in the Pacific, covering 14% of the study population. The seven states are of varying size, geographical location, government structure, and budget.

## Discussion

### Public Information and Communication

Consistent and targeted communication is critical for the acceptability and success of NPIs (Aiello, 2010). Effective communication guides the public, media, and health care providers to respond appropriately to outbreak situations and comply with public health measures. All 62 jurisdictions have developed or are in the process of developing a communications plan. Comprehensive plans should include critical elements such as target audience, goals of the communications, and strategies for reaching the target audience.

PIOs are often given the charge of representing an agency as the spokesperson and serve as the liaison with external stakeholders and lead contact with all media. Studies suggest PIOs are critical to effective dissemination of information by local health departments and are associated with increased information receipt by the general population (Quinn et al., 2013). Sixty respondents identified and trained key staff to serve as spokesperson during a novel influenza threat.

Pandemic influenza is likely to disproportionately affect vulnerable populations; therefore, communication with these groups is particularly important, and strategic planning should consider life circumstances and cultural values when designing communication plans (Blumenshine et al., 2008). Most jurisdictions reported having specific communication plans to reach vulnerable and non–English-speaking populations, but 10 jurisdictions do not have these plans. CDC contracts with a translation service to provide communications materials in multiple languages (CDC, 2017).

### Community Mitigation

All jurisdictions reported they are addressing how to implement NPIs during a pandemic influenza outbreak. Studies based on mathematical models and historical analyses suggest that early implementation of community mitigation measures such as social distancing, school closures, and isolation of sick persons, may be effective in reducing the transmission of influenza virus (Blendon et al., 2008; Ferguson & Cummings, 2006; Markel, 2007).

During an influenza pandemic it may be necessary to temporarily close schools, and knowing who has the legal authority to do so in accordance with what legal mechanisms is crucial to timely execution of the closures (Stern, Cetron, & Markel, 2009). Authorities for closing schools or dismissing students vary widely among states and localities and a patchwork of laws and regulations govern these actions (Stern et al., 2009). The majority of jurisdictions reported either not needing or having the legal authority to close facilities. However, a few jurisdictions did not have such authority, and ill-defined lines of authority are known to cause interagency conflict (Stern et al., 2009) and should be minimized.

Some community mitigation measures, such as staying home from work and school, could cause financial or other burdens. In a nationally representative survey of 1,697 adults, most respondents indicated they would comply with recommendations but would be challenged to do so if their income or job was severely compromised (Blendon et al., 2008). Local prepandemic planning should account for the needs of workers who must rely on hourly wages or do not have paid leave time available.

### Limitations

The results of this study suffer from all traditional limitations of survey research design such as self-reported responses and no external validation. Potential impact of these limitations include inconsistent interpretation of the questions among respondents and a response bias. Future investigations could include an independent review and qualitative data to augment clarity for areas of identified strengths and gaps.

## Conclusion

In the United States, annual vaccination against seasonal influenza is recommended for all persons ≥6 months (Grohskopf et al., 2017). However, the effectiveness of influenza vaccine can vary by season. For the 2017 to 2018 influenza season, the overall estimated effectiveness of the vaccine for preventing influenza virus infection is only 36% (Flannery et al., 2018). Therefore, community level NPIs such as home isolation, school closures, and respiratory etiquette play a critical role in mitigating the spread of disease. Effective, ongoing communication with the public is essential to ensuring wide spread compliance of NPIs, especially among non–English-speaking populations. Planning should also include reaching vulnerable populations and identifying the correct legal authorities for closing schools and canceling mass gatherings. This will ensure all public health programs have planned and prepared for an effective response to a severe influenza pandemic in a community setting. Future evaluation tools developed by the PHEP program should ensure that these critical areas are being met by all 62 jurisdictions.
